# First Reported Case of Integrase Strand Transfer Inhibitor Resistance in Suriname: Unusual Drug Resistance Mutations Following Exposure to Dolutegravir

**DOI:** 10.3390/v17020245

**Published:** 2025-02-11

**Authors:** Rachel C. Sno, Gracia Culbard, Malti R. Adhin

**Affiliations:** 1 “Prof. Dr. Paul C. Flu” Institute for Biomedical Sciences, Kernkampweg 5, Paramaribo, Suriname; rachelcsno@gmail.com; 2 Academic Hospital Suriname, Flustraat 29, Paramaribo, Suriname; 3 Faculty of Medical Sciences, Department of Biochemistry, Anton de Kom Universiteit van Suriname, Kernkampweg 5, Paramaribo, Suriname

**Keywords:** dolutegravir, drug resistance mutations, HIV-1 integrase, INSTI resistance, Suriname

## Abstract

Contemporary ART as Dolutegravir (DTG) has significantly advanced antiretroviral therapy, but relatively few data are available on its impact on the emergence of HIV-1 drug resistance mutations (DRMs). Monitoring the emergence of INSTI-associated DRMs following the introduction of DTG in Suriname will provide general insight and guide national HIV treatment strategies. All people living with HIV (PLHIV) in Suriname, for whom an INSTI drug resistance test was requested between September 2019 and February 2024 (*n* = 20), were included. HIV-1 integrase gene sequences were determined using Sanger sequencing. INSTI-associated mutations were identified using the Stanford HIV Drug Resistance Database program. The majority of the participants (66.7%) harbored HIV-1 subtype B, and 33.3% were B-recombinant forms. In addition to the INSTI wildtype, a strain was revealed carrying E157EQ and one person harbored a highly INSTI-resistant strain (E138K, G140S, Q148H and N155H). The emergence of a highly INSTI-resistant HIV-1 strain in Suriname, with unusual mutations for ART-experienced PLHIV exposed to DTG as the only INSTI, accentuates the need for continuous monitoring of the emergence of INSTI drug resistance mutations, not only to enable timely interventions and optimized treatment outcomes for PLHIV, but also to steer the decision making for ART protocols, especially for second generation INSTIs.

## 1. Introduction

Global human immunodeficiency virus (HIV) strategies aim to end the HIV/AIDS epidemic by 2030, but in developing countries in particular, HIV remains a persistent public health issue, still claiming millions of lives. Antiretroviral therapy (ART) can effectively manage the clinical progression of the disease after HIV infection and impedes the transmission of the virus. By the end of 2023, 30.7 million of the 39.9 million people living with HIV had access to ART [1]. Over the years, different classes of ART have been developed, and in particular, the use of Highly Active Antiretroviral Therapy (HAART), consisting of a combination of three or more drugs from different antiretroviral classes, inhibiting viral reproduction through different mechanisms, signified a major leap in HIV treatment. However, the existing relatively high level of resistance to the first class of antiretroviral drugs used, NRTIs, and the emerging global increase in drug resistance to NNRTI drugs [2], has instigated the development of new drugs as a class of Integrase Strand Transfer Inhibitors (INSTIs), which block the integration of the viral HIV genome into the host human DNA. Raltegravir (RAL) was the first INSTI and was FDA-approved in 2007, initially for clinical use in multidrug resistant treatment-experienced persons, and subsequently in 2009 for multidrug resistant treatment-naïve persons [3]. The second FDA-approved INSTI was Elvitegravir (EVG). Unfortunately, RAL and EVG have a low genetic barrier to resistance and exhibit cross-resistance, thereby limiting their efficacy. Second-generation INSTIs (Dolutegravir (DTG), Bictegravir (BIC) and Cabotegravir (CAB)) have been developed, and DTG first received FDA approval in 2013. Second-generation INSTIs seem to be highly effective in both treatment-naïve and treatment-experienced persons, even in persons harboring multidrug resistance to drugs of the other classes. This high efficacy in combination with a higher genetic barrier to drug resistance and better tolerability steered countries to transition to INSTI-based regimens. Accordingly, Suriname, a small low- and middle-income (LMIC) country, located on the northeastern coast of South America, also introduced the use of RAL in 2016.

HIV is a significant public health issue in Suriname, and with a population of 628,800 and a mortality rate of approximately 43 per 100,000 in 2023, it remains in the top ten of the leading causes of mortality. Free access to HIV testing, counseling, clinical care and treatment is an integral part of the health care system, but in 2023, the national HIV prevalence was still 1.2%, with an annual estimated incidence of 500 infections [4]. A higher HIV prevalence was observed among high-risk populations, including sex workers (SW), men who have sex with men (MSM) and patients with tuberculosis.

As part of the national strategy to manage the impact of HIV, ART is prescribed free of charge to patients based on the test-and-treat strategy, starting ART in PLHIV with a readiness to start treatment, regardless of their CD4 count. In 2023, approximately 46% of PLHIV were receiving ART [4], and 82% of these received DTG, since it is part of the first-line regimen. Although HIV testing and counseling is decentralized, specialized care is only available in the main hospitals in the capital city, Paramaribo. According to national guidelines, clinicians associated with these hospitals request HIV-1 drug resistance testing, including integrase, for PLHIV experiencing treatment failure (clinical or virological) and PLHIV restarting ART after treatment interruption with a switch to a DTG-containing regimen. Baseline drug resistance testing prior to the start of ART is not a prerequisite in the national guidelines.

Following WHO updates [5], Suriname further adapted the national HIV guidelines in 2018 with the use of DTG in combination with an NRTI backbone among people failing a non-DTG-based first-line regimen. Unfortunately, the development and transmission of INSTI-associated drug resistance mutations is inevitable. The current prevalence of INSTI drug resistance ranges from 0.3% in Europe [6] to 1.49% in Guangdong, China [7], in treatment-naïve persons, while reaching a notable 19.6% among highly treatment-experienced patients in Mozambique [8]. Although global resistance data are slowly starting to emerge, information regarding second-generation INSTI resistance remains limited.

The aim of this study was to monitor the emergence of drug resistance mutations associated with INSTI resistance in Suriname. All results of integrase drug resistance testing, performed for people living with HIV (PLHIV) between September 2019 and February 2024, were reviewed.

The first case in Suriname of drug resistance mutations associated with INSTI resistance is presented here in more detail, highlighting rather uncharacteristic mutations.

## 2. Materials and Methods

### 2.1. Study Population

All PLHIV with a request for an INSTI drug resistance test in Suriname in the period from September 2019 to February 2024 were included in this study. Although all hospitals participated in the study and no exclusion criteria were applied, only 20 participants could be included. However, the included samples encompassed all cases with integrase drug resistance testing during the study period. This cohort (*n* = 20) comprised both PLHIV experiencing treatment failure while on a DTG-containing regimen and PLHIV about to switch to a DTG-containing regimen, and none had been exposed to first-generation INSTIs.

### 2.2. Sample and Data Collection

Whole blood samples were collected for HIV-1 drug resistance testing. Basic clinical data were obtained via the clinician as standard supporting information, accompanying each HIV-1 drug resistance request.

### 2.3. RNA Extraction, PCRs and DNA Sequencing

Viral RNA was isolated from 560 µL plasma with the QIAamp Viral RNA kit (QIAGEN Inc., Valencia, CA, USA). PCR amplification of the *pol* gene covering the HIV-1 protease (PR) gene (codons 1–99) and the N-terminal region of the reverse transcriptase (RT) gene (codons 1–300) was performed using customized in-house master-nested real-time polymerase chain reaction (RT-PCR) protocols from the World Health Organization (WHO). PCR amplification of the complete integrase (IN) gene (codons 1–288) was performed using master-nested RT-PCR protocols described by Bessong et al. [9]. Sanger sequencing reactions were conducted with the BigDye^TM^ v3.1 Cycle Sequencing Kit (Applied Biosystems^TM^, Foster City, CA, USA), after PCR product cleanup with ExoSAP-IT^TM^ Express PCR Product Cleanup Reagent (Affymetrix Inc., Santa Clara, CA, USA). Sequencing products were purified with the BigDye Xterminator^TM^ Purification kit (Applied Biosystems^TM^, Foster City, CA, USA) and subsequently sequenced on an ABI 3500XL genetic analyzer (Applied Biosystems^TM^, Foster City, CA, USA).

### 2.4. Sequence Data Analysis

Generated HIV pol sequences were compared with reference sequences to identify mutations and to predict ARV effectiveness, using the Stanford HIV Drug Resistance Database v9.1 [10]. Mutations were defined as differences with regard to the consensus B reference sequence. The impact of drug resistance mutations (DRMs) in each sequence was calculated with the Stanford software, which returns inferred levels of resistance to 25 FDA-approved ARV drugs, including INSTIs. HIV subtyping was carried out with the REGA HIV subtyping tool [11] and recombinants were confirmed with the Recombinant Identification Program (RIP) v3.0 tool [12].

## 3. Results

A total of 22 samples from 20 patients with a male-to-female ratio of 0.54 were included in this study. During the study period, testing was requested thrice for a patient with a complicated history of treatment adherence. The median age of the participants was 42 years, ranging from 12 to 61 years. Among the 20 patients, only 2 were INSTI-naïve at the time of testing and commencing with a DTG-containing regimen. The remaining 18 participants were not exposed to first-generation INSTIs, but had been on DTG for varying periods ranging from 1 month to 4 years at the time of testing. Genotypic drug resistance testing of the integrase gene was successfully performed on 15 samples (68%), while 7 samples were deemed invalid due to insufficient PCR amplification. Since viral load testing in the hospitals was not performed in conjunction with sample collection for genotyping, a low viral load was deemed as a plausible explanation for the suboptimal amplification of samples. In addition, the mismatches of the utilized primers with BD recombinants commonly result in PCR failures, which is especially important in this cohort, with a substantial presence of recombinant viruses.

Subtype determination revealed 66.7% HIV-1 subtype B and 33.3% subtype B recombinant forms. From the 15 obtained genotypic profiles, 87% (*n* = 13) were fully susceptible to INSTIs. One person (Patient 18) harbored a subtype B strain with a naturally occurring polymorphic mutation, E157EQ, while drug resistance mutations associated with resistance to INSTIs were detected in another person (Patient 8). The sequence electropherograms of sections with the important mutations in the strains, derived from the patients not susceptible to INSTI, are provided in Supplementary File S1, Figure S1.

Detailed findings of the patient with an INSTI-resistant strain, who was INSTI-naïve upon the start of a DTG-containing regimen, are presented below, including clinical data and results of the drug resistance tests for mutations associated with resistance to RT and PR.

### Clinical Data of Patient 8

A short description of the patient harboring an HIV strain with drug resistance mutations associated with resistance to INSTIs is presented. From here on, she will be referred to as Patient 8 (this is the number under which she appears in the database). A more detailed description of the patient’s clinical data and treatment history is provided in Supplementary File S1.

In July 2019, a 46-year-old woman with severe proteinuria was admitted to the academic hospital in Paramaribo, the capital of Suriname, and tested positive for HIV. She was initiated on first-line ART (Emtricitabine (FTC)/tenofovir disoproxil fumarate (TDF)/efavirenz (EFV)). In February 2020, she was switched to a second-line ART regimen consisting of FTC/TDF and DTG, after non-adherence prompted by symptoms such as drowsiness and fatigue. In April 2020, a HIV-1 drug resistance test for mutations associated with resistance to RT and PR was requested to assess the impact of the earlier ART used and the suitability of continuance of FTC/TDF in the second-line regimen. After poor adherence was discovered in August 2020, her ART regimen was changed to a third-line regimen, consisting of Lamivudine (3TC), TDF and DTG.

The first HIV-1 drug resistance test, encompassing only the RT and PR genes, revealed one major DRM (G190A) and another accessory mutation (V179E) in the NNRTI class. G190A is associated with intermediate-level resistance to Efavirenz (EFV), which had been used in the period August 2019–February 2020. At this time, no NRTI and PI DRMs were detected. An overview of mutations per drug class is presented in Table 1.

Subtype determination revealed subtype B. In addition to DRMs, we observed 11 and 7 other polymorphisms in PR and RT, respectively. These mutations, classified as non-DRMs, are further referred to as other mutations. The second genotypic drug resistance test, including integrase genotyping, was ordered in May 2022. The other mutations amounted to 12 in PR, and all mutations had already been detected in the first profile in 2020, plus R57K as an additional mutation. Similarly, the pattern of other mutations for the RT gene was identical in both tests. The integrase gene exhibited 13 other mutations (Table 1). The role of the other mutations observed in the IN gene is unclear, as some studies have shown that IN polymorphic substitutions do not compromise the effectiveness of IN-based regimens [13,14], while earlier studies revealed a relationship between IN polymorphic mutations and a decrease in drug susceptibility [15]. For the DRMs, the two RT (NNRTI) mutations detected in the first profile persisted and additional DRMs were observed, associated with high-level resistance to all NRTI drugs except Zidovudine (AZT). Unexpectedly, the genotypic profile also exhibited four major INSTI DRMs (E138K, G140S, Q148H and N155H), associated with high-level resistance to all five currently available INSTI drugs. This multidrug resistance profile prompted a switch to a salvage therapy on July 2022 with Ritonavir-boosted Darunavir (DRV/r) in combination with AZT, resulting in the anticipated undetectable viral load in November 2022.

## 4. Discussion

This study provides insight into the prevalence of integrase drug resistance in Suriname. The results revealed that the majority of successfully amplified samples (87%) remained fully susceptible to INSTIs.

The E157Q mutation observed in one participant is in line with its natural polymorphic character and frequency of occurrence in INSTI-naïve patients. This sole E157Q mutation appears to have minimal impact on INSTI resistance and treatment response.

Patient 8, who was INSTI-naïve upon the start of a dolutegravir-containing regimen, will be discussed in further detail, including results of mutations associated with resistance to RT and PR. Consecutive RT and PR genotyping results of the other mutations from the RT and PR gene implied the evolution of the initial virus strain, rather than re-infection with a new virus strain.

Further examination of the evolution of NRTI/NNRTI DRMs in the consecutive genotyping results revealed the persistence of two NNRTI drug resistance mutations, despite the discontinued use of NNRTIs after the switch to an INSTI regimen combined with two NRTIs. This could be explained by the common observation that NNRTI mutations persist long after the discontinuation of NNRTI therapy [16].

On the other hand, four NRTI DRMs evolved, of which the emergence of the mutations K65KR and M184V after TDF and 3TC use are in line with reports on the evolution of specific mutations associated with the use of these NRTI drugs and earlier results from acquired HIV-1 NRTI drug resistance patterns from Suriname [17,18]. Somewhat unexpected was the presence of two thymidine analog mutations (D67N and K70R), despite the lack of exposure to either of the drugs Zidovudine and Stavudine, which have been reported to select for these two mutations [17].

The presence of these four NRTI mutations in this patient could account for an increased risk of DTG drug resistance, as was demonstrated in a collaborative analysis study with several cohorts, in which the risk for DTG resistance was substantially increased by NRTI resistance [19]. A failing NRTI backbone treatment further supports this hypothesis, as DTG would be rendered as the only functional drug in the combination therapy, implying DTG functional monotherapy, which also increases the risk of INSTI drug resistance.

Next to NRTI/NNRTI DRMs, this HIV strain exhibited four major INSTI drug-resistance-associated mutations; E138K, G140S, Q148H and N155H. E138K is a non-polymorphic mutation in the integrase gene, commonly occurring in patients with exposure to either DTG, RAL, EVG or CAB [20], which is consistent with the patient’s use of DTG.

A recent review of INSTI-naïve PLHIV considered R263K, the so-called signature mutation, and G118R as the most common DRMs, selected by DTG in previously INSTI-naïve persons [21]. Two of the other major DRMs observed in our patient (Q148H and N155H) were also listed as DTG resistance mutations in this review [21], but were less prevalent.

The absence of the signature mutation (R263K) and G118R in our INSTI-naïve, treatment-experienced patient while on a DTG-containing regimen was somewhat unexpected.

The absence of the second-most-common DTG mutation in INSTI-naïve persons (G118R) was probably elicited by the distinct non-overlapping mutational pathways of G118R and N155H/Q148H, resulting in a negative correlation [21]. Furthermore, G118R is more commonly observed in non-B subtypes [22].

The fourth observed DRM (G140S) is a regular non-polymorphic INSTI-associated DRM [21], but is more often observed in RAL-treated patients [23]. Furthermore, the presence of the G140S mutation may account for the absence of the signature mutation (R263K), as these two mutations were reported as incompatible for virus survival [24].

It should be noted that the emergence of G140S, N155H and Q148H, rather than R263K, was also observed in a study, although among humanized mice on DTG monotherapy with no prior ART exposure [25]. These humanized mouse data suggest that these mutations may indeed be selected by DTG in treatment-naïve patients, especially if insufficient antiviral drug pressure is applied.

This study has some limitations. Firstly, the sample size was small, restricting the generalizing of findings to a broader population. However, the 22 included samples encompassed all cases with integrase drug resistance testing among patients with HIV-1 infection in Suriname during the study period. Secondly, the suboptimal amplification of samples, not uncommon for samples with a low viral load, further emphasized the first limitation. Thirdly, possible pre-existing mutations of the initially acquired virus were not determined, since baseline resistance testing is not required in the national treatment guidelines. Fourthly, statements on the evolution of INSTI mutations for Patient 8 were limited by the absence of INSTI genotyping in this patient’s first resistance test, prohibiting a comparison of consecutive INSTI genotyping results. The unavailability of genotyping data from her partner, who was not willing to consent to testing and was not even included in the national unified HIV case registry of Suriname, did not help to elucidate this issue. Furthermore, not all clinical data were available for this patient, therefore prohibiting the precise linking of mutations to specific therapy.

The first data from Suriname of drug resistance associated with INSTI complemented the local HIV-1 drug resistance NRTI/NNRTI mutations data and generated a valuable addition to the still-limited international INSTI data. Overall, INSTI drug resistance among previously INSTI-naïve patients receiving a DTG-based regimen is still rare. The case presented here involves a patient with a highly INSTI-resistant HIV-1 strain, in a country with a fairly recent history of INSTI treatment, and is therefore considered a valuable addition to the global INSTI resistance data. As countries are transitioning to DTG and other second-generation INSTI-based regimens, this case highlights the need for systematic monitoring of the emergence of INSTI drug resistance mutations.

## Figures and Tables

**Table 1:** Drug class mutation profiles of the INSTI-resistant strain.

	Mutations April 2020	Mutations May 2022
Class PI		
DRMs	0	0
Other mutations PI	**11**	**12**
Class NRTI		
DRMs	**0**	**4**
	-	K65KR
	-	D67DN
	-	K70KR
	-	M184V
Class NNRTI		
DRMs	**2**	**2**
	V179E	V179E
	G190A	G190A
Other mutations RT	7	7
Class INSTI		
DRMs	Unknown	**4**
		E138K
		G140S
		Q148H
		N155H
Other mutations IN	Unknown	**13**
		D6E; S17N; M50I; I72IV; L74I; I203M; K215N; I220L; R224RW; D253E; I268L; R269K; D270H

PI, Protease inhibitors; DRM, drug resistance mutation; NRTI, nucleoside reverse transcriptase inhibitors; NNRTI, non-nucleoside reverse transcriptase inhibitors; RT, reverse transcriptase; IN, integrase; INSTI, integrase strand transfer inhibitors.

## Data Availability

Data are contained within the article and Supplementary Materials. Nucleotide sequences were submitted to the GenBank database under accession numbers PQ863263 and PQ863264.

## Supplementary Materials

The following supporting information can be downloaded at: https://www.mdpi.com/article/10.3390/v17020245/s1, File S1: Clinical data of patient 8; Table S1: Treatment history of patient 8; Figure S1: Electropherograms of a section of the *pol* sequence of HIV-1 strains, derived from patients not susceptible to INSTI.
