# Supplementary material for: First Reported Case of Integrase Strand Transfer Inhibitor Resistance in Suriname: Unusual Drug Resistance Mutations Following Exposure to Dolutegravir
Source: Viruses. 2025 Feb 11;17(2):245. doi: 10.3390/v17020245 (PMC11860197; doi:10.3390/v17020245)

Supplementary file S1

Clinical data of patient 8:

Patient history:

In July 2019, a 46-year-old woman was admitted to the academic hospital in Paramaribo, the capital of Suriname, with severe proteinuria and tested positive for HIV.
At the time of diagnosis, only CD4 T-cell count was determined, which was 191 cells/mm^3. One week later, while still admitted to the hospital, HIV viral load (VL) testing was performed (59.000 copies/ml) and she was initiated on first line ART with a single pill (Atripla), comprised of Emtricitabine (FTC)/ tenofovir disoproxil fumarate (TDF)/ efavirenz (EFV). Baseline drug resistance testing prior to the start of ART was not performed, in accordance with local guidelines.
She experienced drowsiness as a side effect from this therapy and suffered weight loss (6 kg within one month) during her hospitalization. After hospital discharge, routine HIV care was provided by her regular physician. Routine doctor's visits revealed an initial significant drop in HIV VL, but in February 2020 the VL had increased to 37.000 copies/ml. The patient admitted to non-adherence only after being confronted with the high VL. Symptoms as drowsiness and fatigue prompted her to discontinue Atripla, but she could not recall the length of the period without medication. A switch to a second-line antiretroviral regimen, consisting of Truvada (FTC/TDF) and DTG, was initiated on February 2020 to circumvent the distressing symptoms.
A HIV-1 drug resistance test for mutations associated with resistance to reverse transcriptase (RT) and protease (PR) was requested to assess the impact of earlier used ART and the continuing presence of FTC/TDF in the second-line regimen.
In august 2020, poor adherence was discovered, due to a suboptimal refill of DTG in the COVID-19 period. In this period, the continuum of care was hindered by the consequences of COVID-19 measures as partial/full shut down of public transportation, limited opening hours of pharmacies and periods of stock out in the pharmacies. The patient could not recall the length of time without DTG while prescribed the combination Truvada with DTG. Subsequently, her ART regimen was changed to a third line regimen, consisting of Lamivudine (3TC), TDF and DTG, resulting in decreased levels of plasma HIV RNA, but still remaining above the undetectable threshold. By May 2022, the plasma HIV RNA levels yet again reached 15.000 copies/mL, triggering a new genotyping test and a subsequent treatment switch to a boosted PI with an INSTI (Ritonavir boosted Atazanavir, r/ATV with DTG).

Supplementary Table S1. Treatment History of patient 8

Period	ART regimen	Viral Load (copies/ml)
July 2019	Tested HIV positive, no treatment	-
August 2019	FTC/TDF/EFV (Atripla)*	59.000
October 2019	FTC/TDF/EFV (Atripla)*	470
February 2020	FTC/TDF/DTG (Truvada / DTG)#	37.000
April 2020[1]	FTC/TDF/DTG (Truvada / DTG)#	
August 2020	TDF/3TC/DTG	-
September 2020	TDF/3TC/DTG	4700
December 2020	TDF/3TC/DTG	110
November 2021	TDF/3TC/DTG*	22.000
February 2022	TDF/3TC/DTG	1.500
May 2022[2]	TDF/3TC/DTG*	15.000
June 2022	ATV/r / DTG	-
July 2022	AZT/DRV/r	-
November 2022		undetectable

ART, Antiretroviral Therapy; FTC, Emtricitabine; TDF, Tenofovir Disoproxil Fumarate; EFV, Efavirenz; DTG, Dolutegravir; 3TC, Lamivudine; ATV, Atazanavir; AZT, Zidovudine; DRV, Darunavir; r, Ritonavir
* Uncertainties in patient statement about the continued use of the ART regimen during this period.
\# Uncertainties of inclusion of DTG in ART regimen.
[1] First drug resistance test ordered (RT and PR)
[2] Second drug resistance test ordered (RT, PR and IN)

Supplementary Figure S1. Electropherograms of a section of the *pol* sequence of HIV-1 strains, derived from patients not susceptible to INSTI.

Codons with relevant mutations are boxed.

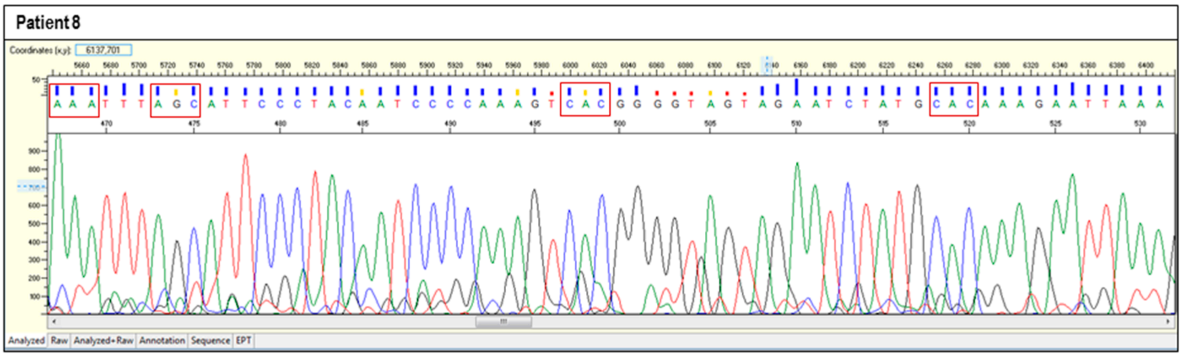

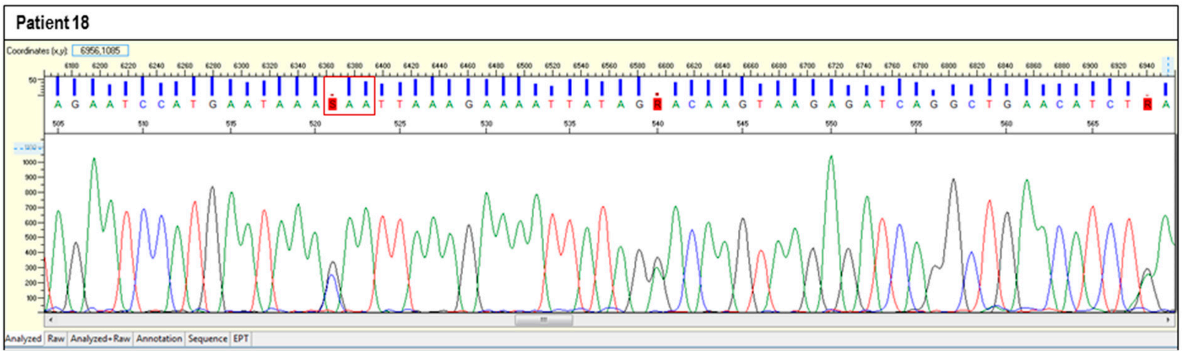

Supplement: Supplementary file 1 [file viruses-17-00245-s001.zip › viruses-3407907-supplementary.pdf]
